# Antihistamine drug terfenadine suppressed the cycle progression of gastric cancer cells by targeting PI3K/AKT/mTOR signaling

**DOI:** 10.3389/fphar.2026.1723444

**Published:** 2026-03-31

**Authors:** Yanping Zhu, Yunhao Ma, Zhenzhen Si, Zhongkun Zhou, Yanan Tian, Yuanchun Zhao, Huanxiang Liu, Hongmei Zhu, Yi Zhang, Jinmei Liu, Peng Chen, Zuoyi Jiao

**Affiliations:** 1 The Second Hospital & Clinical Medical School, Lanzhou University, Lanzhou, China; 2 School of Pharmacy, Lanzhou University, Lanzhou, China; 3 Faculty of Applied Sciences, Macao Polytechnic University, Macao, China; 4 The Second Clinical Medical School, Lanzhou University, Lanzhou, China

**Keywords:** akt, drug repurposing, gastric cancer, PI3K/akt/mtor signaling pathway, terfenadine

## Abstract

**Background:**

Gastric cancer (GC) remains a leading cause of cancer-related mortality worldwide, highlighting the urgent need for effective and accessible therapies. Drug repurposing offers a cost-effective strategy to identify novel candidates from approved drugs. Terfenadine, a classical antihistamine with an established safety profile, has demonstrated antitumor activity in various malignancies; however, its efficacy and mechanism in GC have not been systematically explored. This study investigates the therapeutic potential of terfenadine in GC and elucidates its underlying molecular mechanisms.

**Methods:**

Cytotoxicity was assessed in AGS, HGC27, and MKN45 GC cell lines. Effects on proliferation, colony formation, migration, apoptosis, cell cycle distribution, and mitochondrial membrane potential were examined. Mechanisms were explored through bioinformatics analysis, molecular docking, and Western blotting. Synergy with 5-fluorouracil (5-Fu) was evaluated using checkerboard assays and analyzed by SynergyFinder. *In vivo* efficacy was validated in a patient-derived xenograft (PDX) model.

**Results:**

Terfenadine exhibited potent cytotoxicity against GC cells, with IC_50_ values of 5.14 μM (AGS), 3.95 μM (HGC27), and 5.01 μM (MKN45) at 48 h, demonstrating superior potency compared to 5-Fu in AGS and HGC27 cells. It significantly suppressed colony formation and migration, induced G0/G1 phase arrest *via* downregulation of CDK4/6 and phosphorylated Rb, and promoted mitochondrial apoptosis as evidenced by nuclear condensation and loss of mitochondrial membrane potential. Molecular docking predicted strong binding affinity to AKT (score: 9.09). Western blot analysis revealed that terfenadine treatment reduced the expression of PI3K, total and phosphorylated AKT, and mTOR, indicating modulation of the PI3K/AKT/mTOR pathway. Combination with 5-Fu produced synergistic cytotoxicity (synergy scores >10 b y Loewe and HSA models). In the PDX model, terfenadine (10 mg/kg) significantly suppressed tumor growth, reducing final tumor weight by 41.2% (*p* < 0.001).

**Conclusion:**

This study demonstrates that terfenadine exerts multifaceted antitumor effects in GC through modulation of the PI3K/AKT/mTOR pathway, exhibits synergistic activity with 5-Fu, and shows *in vivo* efficacy in a clinically relevant PDX model. These findings support the repurposing of terfenadine as a promising therapeutic agent for GC.

## 1 Introduction

Gastric cancer (GC) persists as a major global health challenge, ranking fifth in incidence (968,000 new cases) and mortality (660,000 deaths) worldwide in 2022 (Bray et al., 2024). Eastern Asia bears a disproportionate burden, with China alone contributing 358,700 cases (30%–40% global total) (Bray et al., 2024; Mousavi et al., 2025; Han et al., 2024). While diagnostic advancements have improved mechanistic understanding, most patients are diagnosed at an advanced or metastatic stage, leading to a significant reduction in 5-year survival rates (Ma et al., 2023; Smyth et al., 2020; Rawla and Barsouk, 2019). Conventional 5-fluorouracil (5-Fu) and platinum-based chemotherapy encounter two major constraints: dose-limiting myelotoxicity and acquired resistance (Ma et al., 2023; Smyth et al., 2020; Wang et al., 2024; Hou et al., 2017; Yasuda and Wang, 2024; Ouyang et al., 2022). These challenges highlight the urgent need for innovative therapies balancing efficacy with safety—therapeutic repositioning provides a cost-effective paradigm (Xia et al., 2024).

Drug repurposing, also known as drug repositioning, capitalizes on established pharmacokinetic profiles and toxicological data to accelerate development timelines and reduce costs (Xia et al., 2024; Fu et al., 2022). Notable successes include neuropsychiatric drugs: fluoxetine induced apoptosis in GC AGS cells (IC_50_ = 15.00 μM) and enhanced paclitaxel-induced cytotoxicity (Araújo et al., 2022; Po et al., 2020; Khin et al., 2020; Khing et al., 2019), while paroxetine exerted antitumor effects by targeting DNA repair mechanisms (Araújo et al., 2022; Liu et al., 2022). Valproic acid demonstrated antitumor activity in GC AGS cells by targeting the HDAC1/PTEN/AKT signaling cascade (Sun et al., 2020). Additionally, simvastatin, a hydroxymethylglutaryl coenzyme A (HMG-CoA) reductase inhibitor, demonstrated dose-dependent anti-proliferative activity in GC cells (AGS, MKN45, HGC27), effectively suppressing tumor growth (Araújo et al., 2022). Preclinical evidence identified that metformin inhibits GC growth by impairing cell cycle progression (Araújo et al., 2022). These pharmacologically distinct mechanisms collectively broaden the scope of repurposable agents against gastric malignancies.

Among repurposed candidates, H1-histamine receptor (H1R) antagonists exhibit particular promise through pleiotropic antitumor effects (Bakhtiari et al., 2023; H et al., 2024; Trybus and Trybus, 2024; Zhao et al., 2020). Terfenadine, a second-generation H1R antagonist, demonstrates distinct pharmacological advantages over first-generation antihistamines (e.g., diphenhydramine) through reduced blood-brain barrier permeability, minimizing sedative effects while maintaining rapid therapeutic onset and extended half-life (t_1/2_ = 20 h) (Faustino-Rocha et al., 2017; González and Estes, 1998). Beyond its original antiallergic indications, terfenadine showed multi-target activity across malignancies (Trybus and Trybus, 2024; Zhao et al., 2020; Baniya et al., 2024; Fernández-Nogueira et al., 2018; Wang et al., 2014; Ellegaard et al., 2016). Terfenadine induced signal transducer and activator of transcription-3 suppression in colorectal cancer (CRC) *via* mitogen-activated extracellular signal-regulated kinase/extracellular signal-regulated kinase (ERK)/janus tyrosine kinase two axis inhibition (xenograft growth reduction at 10 mg/kg) (Baniya et al., 2024). Terfenadine triggered H1R-dependent apoptosis in hepatocellular carcinoma (HCC) (Trybus and Trybus, 2024; Zhao et al., 2020) and ERK-mediated migration arrest in basal breast cancer (BC) (Fernández-Nogueira et al., 2018). Moreover, terfenadine activated Bak/Mcl-1 cleavage in prostate cancer independent of histamine receptors (Wang et al., 2014). Terfenadine promoted lysosomal death pathways in non-small cell lung cancer (NSCLC) (Ellegaard et al., 2016). Mechanistically, terfenadine coordinated multi-pathway modulation including: calcium (Ca^2+^)/reactive oxygen species-mediated autophagy in melanoma (Nicolau-Galmés et al., 2011; Jangi et al., 2008), and epithelial-mesenchymal transition reversal in chemoresistant NSCLC (An et al., 2017). Despite these advances, clinical translation is constrained by hERG-mediated cardiotoxicity (i.e., blockade of the human Ether-à-go-go-Related Gene potassium channel, which can lead to QT prolongation and potentially fatal arrhythmias) (Olasińska-Wiśniewska et al., 2014). Emerging mitigation strategies, such as low-dose combinatorial regimens that maintain efficacy (An et al., 2017) and nanocarrier-enabled tumor targeting (Baniya et al., 2024; Jin et al., 2022; Fan et al., 2023), are being explored to circumvent this toxicity. Building on these evidences, the present study investigates the therapeutic potential of terfenadine in GC cells, focusing on phosphatidylinositol 3-kinase (PI3K)/protein kinase B (AKT) pathway modulation and apoptosis induction—critical players in gastrointestinal malignancies.

## 2 Materials and methods

### 2.1 Cell culture and drugs

The human GC AGS cells were derived from the American Type Culture Collection (ATCC), AGS (LOT:70012225). The human GC cell lines, HGC27, MKN45 and human gastric mucosa GES-1 cells, were derived from the genetic resource library of our laboratory. AGS, HGC27 and MKN45 cells were cultured in RPMI 1640 (Biological Industries, Israel) supplemented with 10% fetal bovine serum (FBS) (Keygen Biotech, Nanjing, China) and 1% penicillin and streptomycin, and GES-1 was cultured in Dulbecco’s modified Eagle’s medium (DMEM) - high glucose medium containing 10% FBS and 1% penicillin and streptomycin. These cells were cultured at 37 °C in a humidified atmosphere of 5% CO_2_ in air. Terfenadine was obtained from Macklin (Shanghai, China) and 5-Fu was procured from Solarbio (Beijing, China).

### 2.2 Cell viability assay

Cell viability was measured using the MTT (3-(4,5-dimethylthiazol-2-yl)-2,5-diphenyltetrazolium bromide) assay. AGS, HGC27, MKN45 cells were seeded in 96-well plates at a density of 10,000 cells per well in 100 μL of culture medium and allowed to adhere overnight. Cells were then treated with various concentrations of terfenadine (0.10–20.00 μM) or 5-Fu (1.25–100.00 μM for AGS and HGC27, 0.10–20.00 μM for MKN45) for 24, 48, or 72 h. At the end of treatment, 10 μL of MTT solution (5 mg/mL in phosphate-buffered saline) was added to each well (final concentration 0.50 mg/mL), and the plates were incubated at 37 °C for 4 h. The supernatant was carefully removed, and the formazan crystals were dissolved in 150 μL of dimethyl sulfoxide (DMSO). Absorbance was measured at 490 nm using an automated microplate spectrophotometer (Perlong, Beijing, China).

For terfenadine, based on preliminary range-finding experiments and its reported potency in other cancer types, a concentration range of 0.10–20.00 μM was selected to capture the full dose-response curve. For 5-Fu, a broader range of 1.25–100.00 μM was used for AGS and HGC27 cells due to its lower potency in these lines, while a narrower range (0.10–20.00 μM) was applied to MKN45 cells where 5-Fu exhibited higher cytotoxicity. These ranges ensured complete coverage from minimal to maximal effect for each compound and were kept consistent across experiments for each cell line.

### 2.3 Cell cycle assay

Cell cycle was detected by DNA content quantitation assay kit (Solarbio, China). AGS and HGC27 cells were seeded in the 6-well plates at a density of 700,000 cells/well for 24 h. After the treatment of terfenadine of 2.50 μM, 5.00 μM for 24 h, AGS and HGC27 cells were washed in phosphate buffered saline (PBS) twice and fixed in 70% ethanol for 24 h at −20 °C. AGS and HGC27 cells were washed with PBS and then incubated with propidium lodide (PI) with simultaneous treatment of RNase at 37 °C for 30 min. Then the cell cycle distribution and data processing were detected by flow cytometry (Agilent NovoCyte Penteon, Singapore). Fluorescence profiles represented the DNA content of the cells stained by PI. The 24 h time point and concentrations of 2.50 and 5.00 μM were selected because preliminary experiments showed that longer exposure (48 h) led to increased sub-G1 populations (apoptotic cells), which would confound cell cycle distribution analysis. Additionally, treatment with 10.00 μM terfenadine for 24 h induced excessive cytotoxicity, resulting in a large sub-G1 peak that made reliable cell cycle phase quantification impossible. Therefore, these conditions were chosen to optimally capture early cell cycle perturbations before the onset of widespread apoptosis.

For comparison with conventional chemotherapy, cells were treated with 5-Fu under optimized conditions. Based on preliminary dose-response experiments, 5.00 μM 5-Fu was selected for cell cycle analysis (24 h treatment) to avoid excessive cytotoxicity that would compromise cell cycle distribution analysis.

### 2.4 Cell apoptosis assay

Cell apoptosis was quantified using an Annexin V Alexa Fluor488/PI cell apoptosis detection kit (Solarbio, Cat# CA1040). In brief, AGS and HGC27 cells were plated in the 6-well plates at a density 700,000 cells/well. Following a 24 h incubation period, AGS and HGC27 cells were exposed to terfenadine at varying concentrations (2.50 μM, 5.00 μM and 10.00 μM) for 48 h. AGS and HGC27 cells were stained with Annexin V/Alexa Fluor 488 and PI solution for 10 min in dark. Apoptosis was analyzed by flow cytometry (Agilent NovoCyte Penteon, Singapore). The 48 h time point was chosen because apoptosis is a later consequence of drug-induced stress, and preliminary time-course experiments indicated that significant apoptotic populations were only detectable after 48 h of treatment. The concentration range of 2.50–10.00 μM was used to capture the full dose-dependent induction of apoptosis, including both early and late stages.

For apoptosis assessment, 10.00 μM 5-Fu was used (48 h treatment); this concentration was chosen to match the highest concentration of terfenadine (10.00 μM) used in the apoptosis assay, enabling a direct comparison of pro-apoptotic efficacy.

### 2.5 Colony formation assay

The GC AGS or HGC27 cells were seeded in 24-well plates at 500 per well and cultured (5% CO_2_, 37 °C) for 24 h. Then 100 μL of terfenadine solutions with the different concentrations of 0.25 μM, 0.50 μM, 1.25 μM, 2.50 μM were added to the wells and the cells were cultured for another 8–10 days. Finally, the cells were washed with PBS, fixed with 4% paraformaldehyde solution for 40 min, then stained with crystal violet dye solution (0.1%) (Solarbio, Cat# G1061) for 20 min, and finally counted for analysis.

### 2.6 Transwell migration assay

Cell migration was assessed using Transwell inserts. The pore size of the Transwell membrane was 8.00 μm. To prepare for the transwell migration assay, 30,000 AGS or HGC27 cells of 150 μL suspension containing 1% FBS and terfenadine at 0.25 μM, 0.50 μM and 1.25 μM were added to the transwell upper chamber. 600 μL medium containing 20% FBS was added to each bottom chamber. After incubation of 48 h at 37 °C in a 5% CO_2_ atmosphere, the chamber was washed with PBS, fixed with 4% paraformaldehyde for 40 min and then stained with 0.1% crystal violet dye solution for 20 min at room temperature. The cells above the Transwell membrane were wiped with a cotton swab and the migratory cells of the lower chamber were photographed by optical microscope (Novel Optics, Zhejiang, China). The results were statistically analyzed by ImageJ. Each data with SD was acquired from three replications.

### 2.7 DAPI staining and mitochondrial membrane potential (MMP) assay

For nuclear morphology analysis, AGS and HGC27 cells were seeded in 24-well plates at a density of 150,000 cells/well and cultured for 24 h. Cells were then treated with terfenadine at indicated concentrations (2.50 μM, 5.00 μM, and 10.00 μM) for 48 h. After treatment, cells were fixed with 4% paraformaldehyde for 15 min at room temperature and stained with 4′,6-diamidino-2-phenylindole (DAPI) staining solution (Coolaber, Beijing, China) for 10 min in the dark. Nuclear morphology was observed and photographed using a fluorescence microscope (Olympus BX53+DP74, Japan). Apoptotic cells were identified by the presence of condensed or fragmented nuclei.

Mitochondrial membrane potential (ΔΨm) was assessed using a Tetramethylrhodamine ethyl ester (TMRE) Assay Kit (Beyotime Biotech, China). AGS and HGC27 cells were seeded in 24-well plates at a density of 150,000 cells/well, cultured for 24 h, and then treated with terfenadine (2.50 μM, 5.00 μM, and 10.00 μM) for 48 h. As a positive control for mitochondrial depolarization, cells were treated with 10.00 μM Carbonyl cyanide 3-chlorophenylhydrazone (CCCP) for 20 min at 37 °C prior to staining. Cells were then incubated with TMRE staining solution according to the manufacturer’s instructions. Red fluorescence was visualized using a fluorescence microscope (Olympus BX53+DP74, Japan), and a decrease in fluorescence intensity indicated loss of MMP. The mean fluorescence intensity was quantified using ImageJ software from at least three random fields per well. All experiments were performed in triplicate.

### 2.8 Molecular docking

The crystal structures of CDK4 protein (PDB: 3g33) bound to molecules in docking were obtained from the protein databank (PDB, http://www.rcsb.org). Molecular docking was performed by Schrödinger 10.1 software (Schrödinger, United States). First, we removed the complex protein structure of water of crystal, side chain and hydrogen atom, and processed protein with minimal energy. The center of small molecule binding to the protein was used as the docking site of terfenadine and the proteins for analysis.

### 2.9 Bioinformatics analysis

Analysis of AKT protein expression across GC subtypes: To evaluate AKT protein expression in different molecular subtypes of GC, we utilized the UALCAN web portal (http://ualcan.path.uab.edu). The analysis was performed using the Clinical Proteomic Tumor Analysis Consortium (CPTAC) dataset, which contains mass spectrometry-based proteomic data for GC samples. Samples were stratified by microsatellite instability (MSI) status into MSI-high (MSI-H, n = 4) and MSI-low (MSI-L, n = 76) groups. Protein expression values were presented as Z-values, and the statistical significance of the difference between the two groups was assessed using the default parameters implemented in UALCAN. The results are displayed as box plots in Figure 5C.

AKT1 mRNA expression was compared between gastric adenocarcinoma (STAD, n = 408) and normal tissues (TCGA normal, n = 36; combined TCGA + GTEx normal, n = 211) using GEPIA2 (http://gepia2.cancer-pku.cn/), with expression values log2-transformed (TPM+1). The prognostic value of AKT1 was assessed using the Kaplan–Meier Plotter (https://kmplot.com/analysis/). Patients were stratified by the auto-select best cutoff, and overall survival was compared using the log-rank test, with hazard ratio (HR) calculated by a Cox model.

Single-cell analysis: Single-cell RNA sequencing data of GC were obtained from the ASSISTANT for Clinical Bioinformatics database (https://www.aclbi.com/staic/index.html#/). Cell clustering was performed using the t-SNE algorithm (perplexity = 30, max iterations = 1000) with default parameters. Cell types were annotated based on canonical marker genes. AKT1 expression patterns across cell populations were visualized using feature plots (log-transformed, color-coded from purple/low to yellow/high) and quantified as the mean expression level per cell type in a bar plot.

### 2.10 Western blotting

The AGS and HGC27 cells were seeded in a 6-well plate with 600,000 cells/well. After the treatment of terfenadine of 5 μM for 48 h, the cells were lysed by the mixture of cell lysates buffer (Solarbio, Cat# R0010) with 1% phenylmethanesulfonyl fluoride (PMSF) and phosphatase inhibitor buffer on the ice. The mixture of cell lysates and loading buffer was boiled and loaded into sodium dodecyl sulfate polyacrylamide gel for electrophoresis. The protein was transferred to polyvinylidene difluoride (PVDF) membrane (Biosharp, MerckMillipore ISEQ00010) and blocked with 5% fat-free dry milk in Tris-buffered saline Tween-20 (TBST) at room temperature for 1–2 h. After the incubation with primary antibody overnight at 4 °C, the PVDF membrane was washed with TBST and probed with a corresponding horseradish peroxidase-conjugated secondary antibody for 1–2 h at room temperature. Signals on the PVDF membranes were detected by Tanon imaging system (Tanon Science & Technology Co., Ltd.) after Enhanced chemiluminescence (ECL) reagent (New Cell & Molecular Biotech Co., Ltd., Cat# P10200) treatment. The primary antibodies used included Rb (1:1000 dilution), p-Rb (1:1000 dilution), CDK4 (1:1000 dilution), CDK6 (1:1000 dilution) and GAPDH (1:1000 dilution) from Sangon (Shanghai, China), PI3K (1:1000 dilution) from Affinity Biosciences (California, United States), AKT (1:1000 dilution), p-AKT (1:1000 dilution), mTOR (1:1000 dilution), p-mTOR (1:1000 dilution) from Immunoway Biotechnology (Texas, United States).

Western blot band intensities were quantified using ImageJ software. For each protein of interest, the raw integrated density of the specific band was measured after background subtraction. To minimize inter-gel variability, all samples within each comparison (control vs. treatment) were run on the same gel. For normalization, the signal intensity of each target protein was first normalized to the corresponding GAPDH loading control from the same membrane. For phosphorylated proteins (p-Rb, p-AKT, p-mTOR), the normalized values were further used to calculate the ratio of phosphorylated to total protein (p-Rb/total Rb, p-AKT/total AKT, p-mTOR/total mTOR) using values obtained from the same membrane whenever possible. In cases where phosphorylated and total proteins were detected on separate membranes due to antibody compatibility, we ensured that the samples for both membranes were from the same biological replicates and run under identical conditions. All experiments were performed in triplicate with independent biological replicates, and representative blots are shown. Quantitative data are presented as mean ± SD of three independent experiments.

### 2.11 Combination treatment and synergy analysis

The inhibitory effects of single drugs and their combinations on AGS and HGC27 cell viability were evaluated using the MTT assay. Cells were seeded in 96-well plates at 5000 cells per well, treated with serial dilutions of terfenadine (0–10.00 μM), 5-Fu (0–10.00 μM), or their combinations for 48 h, followed by incubation with 10 μL MTT solution (5.00 mg/mL) for 4 h. The formazan crystals were dissolved in 100 μL DMSO, and absorbance was measured at 490 nm. Inhibition rates were calculated relative to untreated controls. A checkerboard matrix design was employed, generating a 7 × 7 concentration matrix (terfenadine: 0, 0.25, 0.50, 1.25, 2.50, 5.00, 10.00 μM, 5-Fu: 0, 0.25, 0.50, 1.25, 2.50, 5.00, 10.00 μM). Each concentration combination was tested in three independent experiments with three technical replicates.

Drug combination synergy was quantified using the SynergyFinder 3.0 web application (https://synergyfinder.fimm.fi). Data were formatted as a seven-column table (PairIndex, Drug1, Drug2, Conc1, Conc2, Response, ConcUnit) and analyzed with the Loewe and HSA models. Baseline correction was applied, and synergy scores were interpreted according to platform guidelines (scores < −10: antagonism, −10 to 10: additivity, >10: synergy). The overall synergy score (δ-score) and the most synergistic area (MSA) score were derived for each model. Visualization outputs including synergy heatmaps and 3D synergy surface plots were generated directly from the platform.

### 2.12 Patient-derived xenograft model

To evaluate the *in vivo* antitumor efficacy of terfenadine, a patient-derived xenograft (PDX) model of GC was established. Fresh tumor specimens obtained from GC patients were minced into approximately 10 mm^3^ fragments, embedded in high-concentration Matrigel (Corning, #354248), and subcutaneously implanted into the flank of 6-week-old female NOD/ShiLtJGpt-Prkdc^*em26Cd52*^Il2rg^*em26Cd22*^/Gpt mice (Gempharmatech, #T001475). When tumors reached approximately 1000 mm^3^, they were excised and re-transplanted into naive mice; this process was repeated until the third passage (P3). For efficacy evaluation, mice bearing P3 tumors with a volume of approximately 150 mm^3^ were randomly assigned to receive either terfenadine (10 mg/kg, administered intraperitoneally once daily) or an equal volume of vehicle (5% DMSO +30% PEG300 + 65% saline). Tumor dimensions were measured every 3 days using a digital caliper, and tumor volume was calculated as length × width^2^ × 0.5. Relative tumor volume was normalized to the initial measurement. Animals were euthanized when tumor volume exceeded 1500 mm^3^ or at the end of the study, and tumors were excised and weighed. All animal procedures were approved by the Medical Ethics Committee of Lanzhou University Second Hospital.

### 2.13 Statistical analysis

The data were expressed as the mean ± SD and significant differences were calculated by t-test. *P values* < 0.05, *p values* < 0.01 and *p values* < 0.001 were considered to be significantly different. All data were made with SPSS 22.0 and GraphPad Prism Software 8.0 and AI 2020.

## 3 Results

### 3.1 The cytotoxicity evaluation of terfenadine in different GC cells

**FIGURE 1:**
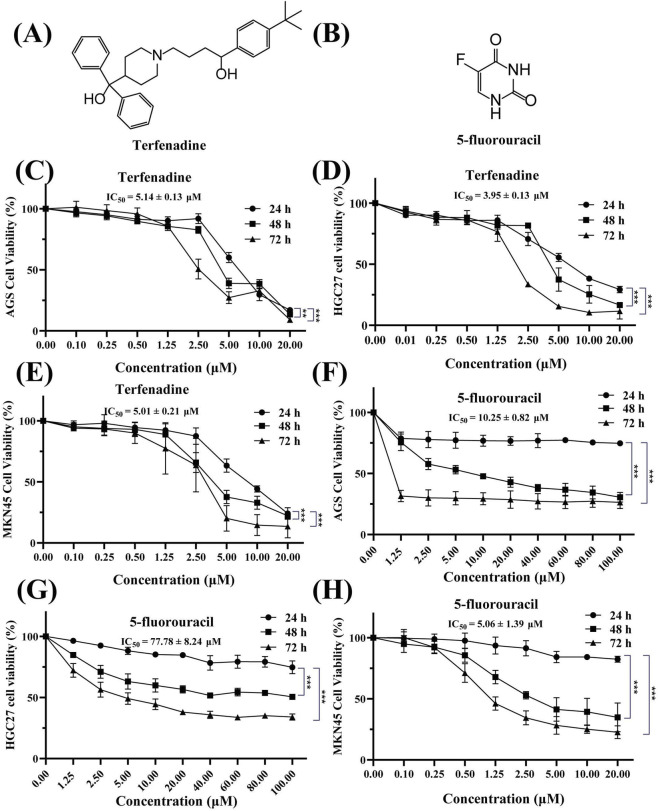
The structure of terfenadine and 5-fluorouracil and their cytotoxic effects in different gastric cancer cells **(A,B)** Chemical structures of terfenadine **(A)** and 5-fluorouracil (5-Fu, **(B,C–E)** Cytotoxicity of terfenadine in AGS **(C)**, HGC27 **(D)**, and MKN45 **(E)** cells. Cells were treated with increasing concentrations of terfenadine (0.10–20.00 μM) for 24, 48, and 72 h, and cell viability was measured by MTT assay **(F,G)** Cytotoxicity of 5-Fu in AGS **(F)** and HGC27 **(G)** cells treated with 1.25–100.00 μM for 24, 48, and 72 h **(H)** Cytotoxicity of 5-Fu in MKN45 cells treated with 0.10–20.00 μM for 24, 48, and 72 h. Data are presented as mean ± SD of three independent experiments. IC_50_ values were calculated by nonlinear regression using GraphPad Prism 8.0. *p < 0.05, **p < 0.01, ***p < 0.001 compared to the negative control group.

The chemical structures of terfenadine and 5-Fu are illustrated in Figures 1A,B, respectively. To assess the anticancer potential of terfenadine, dose-response assays were performed in 3 GC cell lines: AGS (moderately differentiated adenocarcinoma), HGC27 (poorly differentiated metastatic carcinoma), and MKN45 (undifferentiated carcinoma). Terfenadine exhibited significant dose- and time-dependent growth inhibition in AGS, HGC27, and MKN45 GC cell lines (Figures 1C–E). After 48 h treatment, the IC_50_ values were 5.14 ± 0.13 μM (AGS), 3.95 ± 0.13 μM (HGC27), and 5.01 ± 0.21 μM (MKN45), respectively (Figures 1C–E). Comparative analysis with 5-Fu revealed distinct cytotoxic profiles. At 48 h, the IC_50_ values of 5-Fu were 10.25 ± 0.82 μM (AGS), 77.78 ± 8.24 μM (HGC27), and 5.06 ± 1.39 μM (MKN45) (Figures 1F–H). The concentration ranges for terfenadine (0.10–20.00 μM) and 5-Fu (1.25–100.00 μM for AGS and HGC27, 0.10–20.00 μM for MKN45) were selected to optimally capture the full dose-response profile of each compound based on their distinct potency and solubility characteristics. Notably, terfenadine demonstrated ≥ 2-fold higher potency than 5-Fu in AGS and HGC27 cells. Based on this enhanced efficacy, AGS and HGC27 cells were selected for subsequent mechanistic studies.

### 3.2 Terfenadine induced apoptosis and arrested the cells cycle of GC AGS and HGC27 cells

**FIGURE 2:**
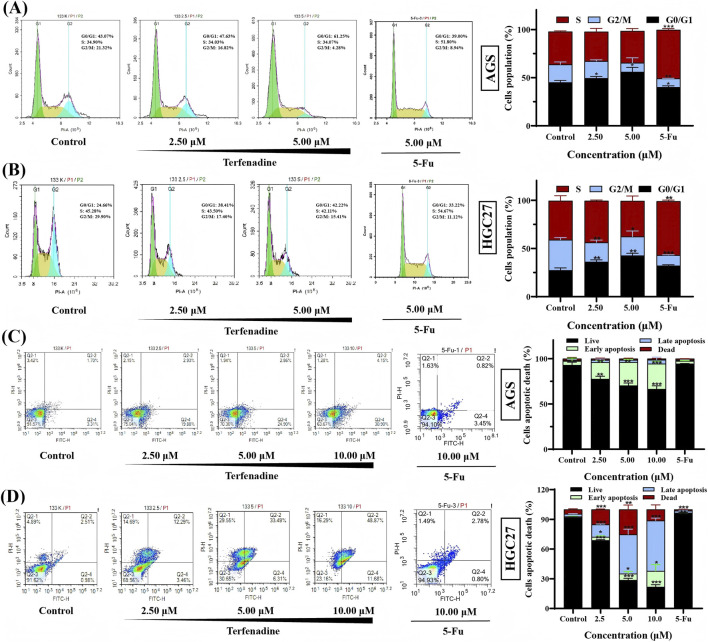
Terfenadine arrested cell cycle and induced cell apoptosis in AGS and HGC27 cells **(A,B)** Terfenadine (2.50 μΜ and 5.00 μΜ) arrested AGS **(A)** and HGC27 **(B)** cells at G0/G1 phase after treatment for 24 h **(C,D)** Terfenadine (2.50 μΜ, 5.00 μΜ and 10.00 μΜ) induced apoptosis of gastric cancer AGS **(C)** and HGC27 **(D)** cells after treatment for 48 h. Data are presented as mean ± SD of three independent experiments. *p < 0.05, **p < 0.01, ***p < 0.001 compared to the negative control group.

Flow cytometry analysis revealed that terfenadine treatment (2.50–5.00 μM, 24 h) triggered dose-dependent G0/G1 phase arrest in AGS and HGC27 cells (Figures 2A,B). In AGS cells, the proportion of G0/G1 phase cells increased from 45.17% ± 1.97% (control) to 56.08% ± 4.66% (5.00 μM terfenadine) (*p* < 0.05). Similarly, HGC27 cells exhibited a marked increase in G0/G1 phase population from 27.31% ± 2.56%–42.64% ± 2.39% at 5.00 μM (*p* < 0.01). G0/G1 phase blockade by terfenadine may sensitize GC cells to DNA-damaging agents by prolonging exposure to genotoxic stress during DNA replication checkpoints. This synergistic effect warrants further investigation in combination therapies.

Terfenadine induced concentration-dependent apoptosis in GC cells. Flow cytometry analysis with Annexin V/PI staining revealed distinct apoptotic populations in AGS and HGC27 cells following terfenadine treatment (2.50–10.00 μM, 48 h). The early apoptosis (Annexin V^+^/PI^−^) proportion increased from 4.41% ± 3.32% (control) to 28.04% ± 2.56% (10.00 μM) in AGS cells (*p* < 0.001), and from 0.92% ± 0.16%–16.02% ± 6.51% in HGC27 cells (*p* < 0.05). The late apoptosis (Annexin V^+^/PI^+^) proportion rose from 0.78% ± 0.81%–4.31% ± 2.98% in AGS cells (*p* < 0.05), and from 2.64% ± 0.22%–50.92% ± 2.49% in HGC27 cells at 10.00 μM (*p* < 0.001) (Figures 2C,D). Notably, HGC27 cells exhibited a non-linear dose-response at higher concentrations, with 5.00 μM terfenadine inducing greater late apoptosis than 10.00 μM (Figure 2D). This divergence may reflect saturation of apoptotic pathways or activation of necrosis at elevated doses. Terfenadine simultaneously induced G0/G1 phase arrest (Figures 2A,B) and apoptotic commitment in GC cells, suggesting a dual mechanism to suppress proliferation and promote cell death.

To benchmark terfenadine’s effects, we evaluated the cell cycle and apoptosis profiles of the conventional chemotherapeutic agent 5-Fu under optimized conditions (Figure 2).

For cell cycle analysis (5.00 μM, 24 h), 5-Fu consistently induced S phase arrest in both cell lines. In AGS cells, the S phase fraction increased from 34.14% ± 0.68%–50.60% ± 1.36% (*p* < 0.01), with a concomitant reduction in G0/G1 and G2/M phases. A more pronounced S phase accumulation was observed in HGC27 cells (40.51% ± 5.17%–55.98% ± 1.20%, *p* < 0.01), accompanied by a sharp decrease in G2/M phase (from 31.74% ± 2.21%–10.85% ± 0.38%, *p* < 0.001). This pattern aligns with 5-Fu’s mechanism as an antimetabolite and, in HGC27 cells, reflects its p53-null status lacking a functional G1 checkpoint.

For apoptosis assessment (10.00 μM, 48 h)—a concentration matching the highest terfenadine dose used—5-Fu induced minimal apoptosis in both cell lines. Total apoptosis (early + late) remained unchanged in AGS cells (control: 5.19% ± 3.32% vs. 5-Fu: 4.04% ± 0.35%, *p* > 0.05) and in HGC27 cells (control: 3.56% ± 0.38% vs. 5-Fu: 2.77% ± 0.55%, *p* > 0.05). These results demonstrate that at 10.00 μM, 5-Fu acts primarily as a cytostatic agent causing cell cycle arrest, with negligible pro-apoptotic activity. This contrasts sharply with the potent apoptosis induced by terfenadine at the same concentration (Figures 2C,D).

### 3.3 Terfenadine suppressed the proliferation and migration of GC AGS and HGC27 cells

**FIGURE 3:**
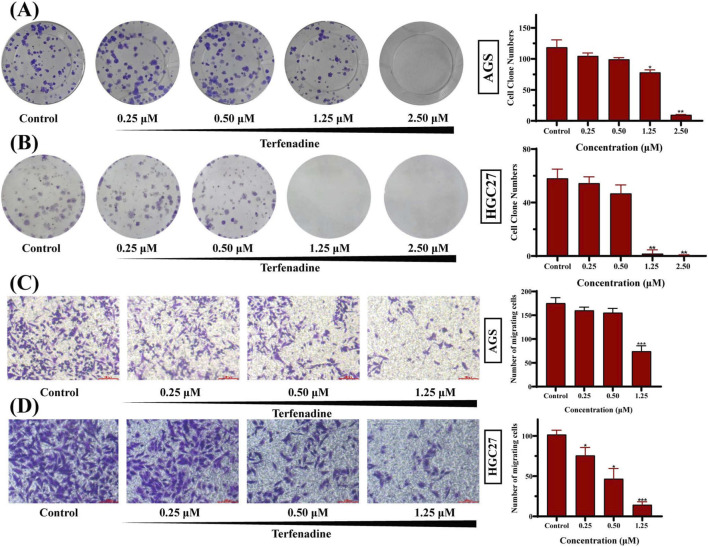
Terfenadine inhibits colony formation and migration of AGS and HGC27 cells **(A,B)** Terfenadine suppresses the colony formation of gastric cancer cells. AGS **(A)** and HGC27 **(B)** cells were treated with the indicated concentrations of terfenadine (0.25, 0.50, 1.25, and 2.50 μM) for 8–10 days. Colony formation was assessed by crystal violet staining. Representative images (left panels) and quantitative analysis of colony numbers (right panels) are shown. Data are presented as mean ± SD of three independent experiments. *p < 0.05, **p < 0.01 compared to the negative control group **(C,D)** Terfenadine inhibits the migration of gastric cancer cells. AGS **(C)** and HGC27 **(D)** cells were treated with terfenadine (0.25, 0.50, and 1.25 μM) for 48 h in Transwell chambers. Migrated cells were stained with crystal violet and photographed (left panels). Quantification of migrated cell numbers is shown in the right panels. Data are presented as mean ± SD of three independent experiments. *p < 0.05, **p < 0.01, ***p < 0.001 compared to the negative control group.

Terfenadine inhibited the proliferation of GC cells in a concentration-dependent manner. Terfenadine (0.25–2.50 μM) significantly reduced colony formation in AGS and HGC27 cells (Figures 3A,B). At 2.50 μM, colony counts decreased from 119 ± 12 (control) to 10 ± 1 in AGS cells (*p* < 0.01), and from 58 ± 7 to 0 in HGC27 cells (*p* < 0.01), demonstrating near-complete suppression of proliferative capacity. In Transwell migration assays, terfenadine (0.25–1.25 μM, 48 h) markedly impaired GC cell motility. Migrated cell numbers decreased from 175 ± 12 (control) to 74 ± 12 in AGS cells (*p* < 0.001), and from 102 ± 6 to 14 ± 4 in HGC27 cells (*p* < 0.001) at 1.25 μM (Figures 3C,D).

### 3.4 Terfenadine induced apoptosis by decreasing the MMP of GC AGS and HGC27 cells

**FIGURE 4:**
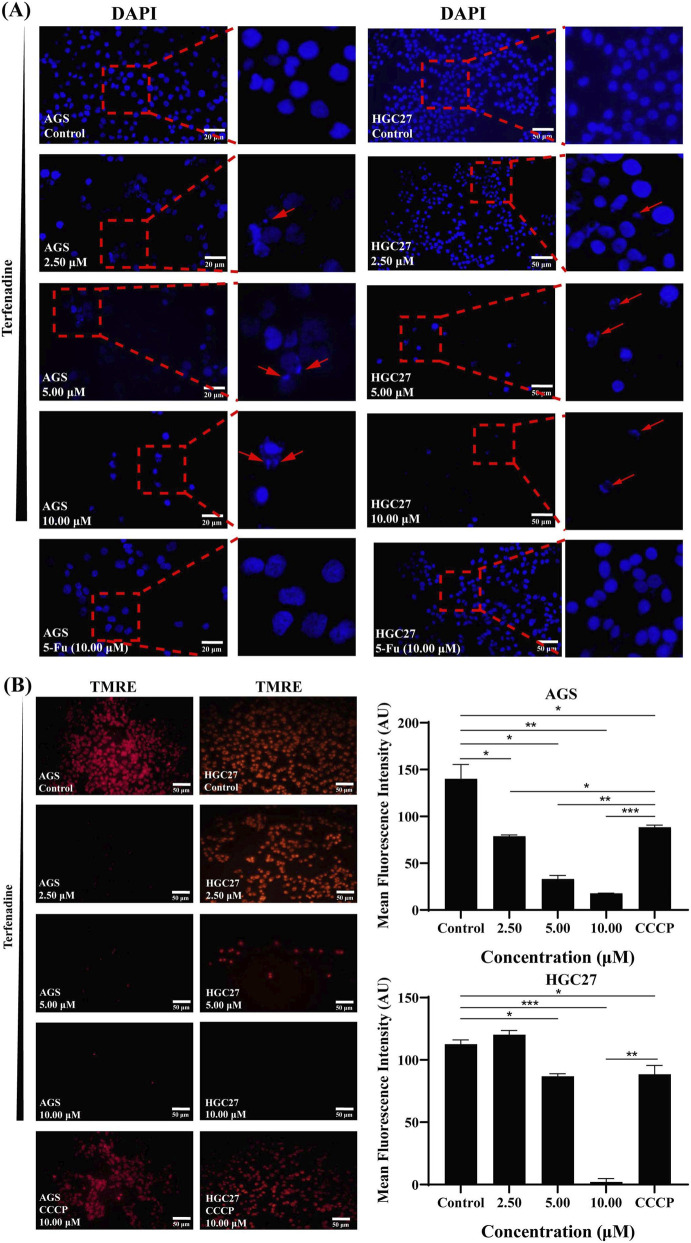
Terfenadine induces apoptosis and reduces mitochondrial membrane potential in AGS and HGC27 cells. Cells were treated with terfenadine at the indicated concentrations (2.50, 5.00, and 10.00 μM) for 48 h. Carbonyl cyanide 3-chlorophenylhydrazone (CCCP, 10 μM) was used as a positive control for mitochondrial membrane potential depolarization **(A)** Terfenadine induces nuclear condensation and apoptotic body formation. Representative fluorescence microscopy images of AGS and HGC27 cells stained with DAPI (blue) are shown. Red arrows indicate cells with condensed or fragmented nuclei, characteristic of apoptosis **(B)** Terfenadine causes loss of mitochondrial membrane potential (ΔΨm). Cells were stained with TMRE (red fluorescence), and images were captured by fluorescence microscopy. Representative images (left panels) and quantitative analysis of mean fluorescence intensity (right panels) are presented. Data are expressed as mean ± SD of three independent experiments. *p < 0.05, **p < 0.01, ***p < 0.001 compared to the untreated control group.

Terfenadine induced mitochondrial-dependent apoptosis in GC cells. Fluorescence microscopy revealed terfenadine-treated AGS and HGC27 cells exhibited nuclear condensation and apoptotic body formation, contrasting with 5-Fu-treated cells that maintained relatively intact nuclei (Figure 4A). Terfenadine induced MMP collapse in GC cells. In AGS cells, the mean intensity of red fluorescence was significantly decreased from 140.11 ± 15.30 AU (control) to 17.67 ± 0.47 AU (10 μM) and 88.36 ± 2.46 AU (CCCP, 10 μM), compared to control (*p* < 0.01). In HGC27 cells, the mean intensity of red fluorescence was significantly decreased from 112.62 ± 3.48 AU (control) to 2.00 ± 2.83 AU (10 μM) and 88.38 ± 7.17 AU (CCCP, 10 μM), compared to control (*p* < 0.001) (Figure 4B).

### 3.5 AKT is a potential target of terfenadine in inhibiting the growth of GC AGS and HGC27 cells

**FIGURE 5:**
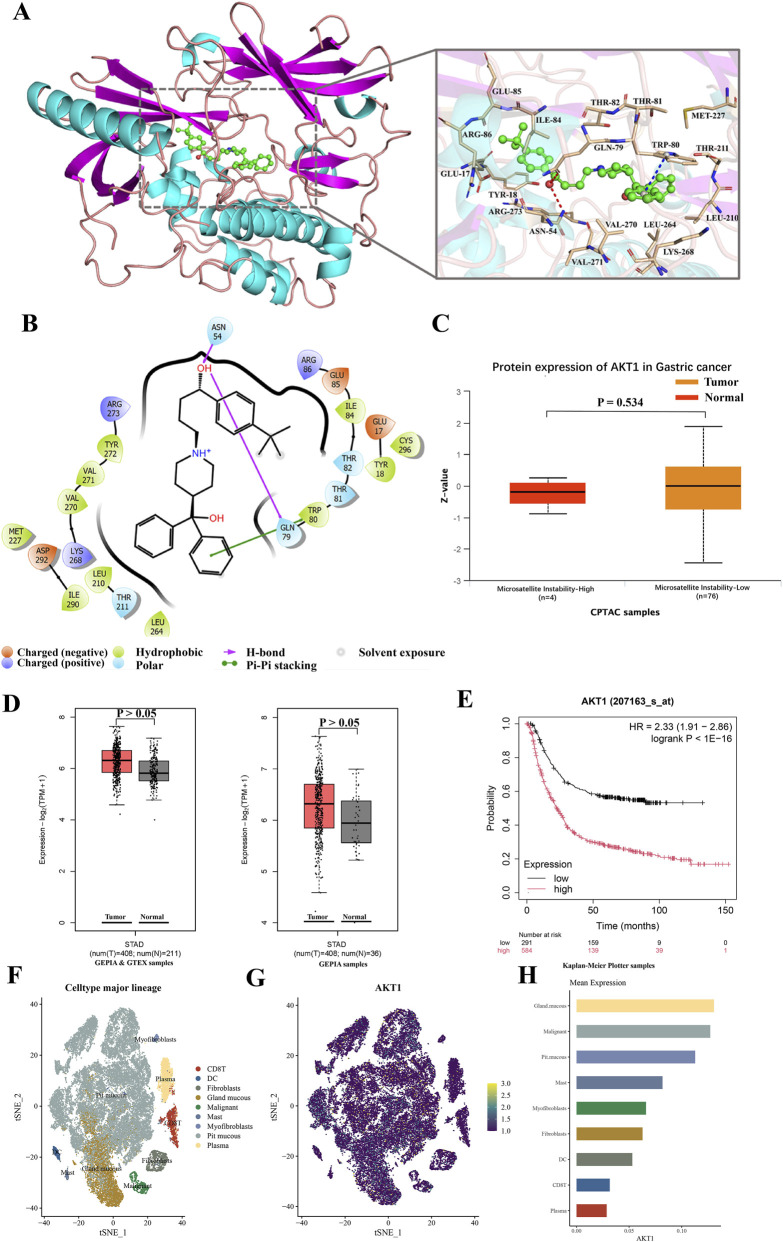
Molecular docking of terfenadine with AKT and bioinformatics analysis of AKT expression in GC **(A)** Detailed 3D interaction diagram of terfenadine binding to the AKT protein (PDB: 6hhf) **(B)** 2D diagram illustrating the key interactions between terfenadine and amino acid residues of AKT **(C)** Analysis of AKT protein expression in GC tissues stratified by microsatellite instability (MSI) status. Box plots show Z-values of AKT protein expression in MSI-high (MSI-H, n = 4) and MSI-low (MSI-L, n = 76) tumors from the CPTAC dataset accessed *via* UALCAN. No significant difference was observed between the two groups (*p* = 0.534) **(D)** GEPIA2 analysis of AKT1 mRNA expression in GC (n = 408) vs. normal tissues (TCGA, n = 36; TCGA + GTEx, n = 211). Neither comparison revealed a statistically significant difference (*p* > 0.05) **(E)** Kaplan-Meier survival analysis of GC patients stratified by AKT1 mRNA expression (low, n = 291; high, n = 584) using the Kaplan-Meier Plotter. HR, hazard ratio **(F–H)** Single-cell RNA sequencing analysis of *AKT* gene expression in different cell subpopulations within GC tissue, derived from the ASSISTANT for Clinical Bioinformatics database **(F)** t-SNE plot of single-cell transcriptomes from GC, colored by major cell lineages **(G)** Feature plot showing AKT1 expression levels (log-transformed, purple/low to yellow/high) projected onto the t-SNE map **(H)** Bar plot quantifying mean AKT1 expression across the indicated cell lineages.

To identify the potential molecular target of terfenadine in GC cells, we performed molecular docking experiments with a series of proteins involved in cell cycle regulation, apoptosis, and signaling pathways (docking scores are summarized in Supplementary Table S1). Among these, terfenadine exhibited a strong binding affinity to AKT (PDB: 6hhf), with a docking score of −9.09. Detailed analysis of the docking pose predicted that the hydroxyl group of terfenadine may form two hydrogen bonds with Asn54 and Gln79 of AKT, potentially enhancing binding stability. Additionally, the benzene ring of terfenadine appeared to engage in π-π stacking with Trp80, and electrostatic interactions were predicted with several residues, including Lys268, Arg273, Arg86, Asp292, Glu17, and Glu85 (Figures 5A,B). These interactions collectively suggest that AKT is a plausible direct target of terfenadine.

Quantitative proteomic analysis of the CPTAC dataset *via* UALCAN revealed that AKT protein levels were comparable between MSI-H (n = 4) and MSI-L (n = 76) tumors (*p* = 0.534), indicating that AKT expression is independent of MSI status and is a common feature across GC subtypes (Figure 5C). At the mRNA level, GEPIA2 analysis showed no significant difference in AKT1 expression between GC tissues (n = 408) and normal gastric tissues, regardless of whether the normal control group was derived from TCGA alone (n = 36) or combined TCGA and GTEx datasets (n = 211) (both *p* > 0.05, Figure 5D). Despite the lack of difference in mean mRNA levels between tumor and normal tissues, survival analysis using the Kaplan-Meier Plotter database demonstrated that high AKT1 mRNA expression within the GC patient population was strongly associated with poorer overall survival (HR = 2.33, 95% CI: 1.91–2.86, log-rank *p* < 1E-16, Figure 5E). This finding underscores the clinical significance of AKT expression and its potential as a prognostic biomarker.

To further refine the cellular context of AKT expression within the gastric tumor microenvironment, we analyzed single-cell RNA sequencing data. The t-SNE plot revealed a clear separation of major cell lineages, including epithelial cells (pit mucous cells, gland mucous cells, malignant cells), stromal cells (fibroblasts, myofibroblasts), and immune subsets (CD8^+^ T cells, dendritic cells, mast cells, plasma cells) (Figure 5F). A feature plot projecting AKT1 expression onto this cellular map showed that higher expression levels (blue to yellow dots) were predominantly observed in subsets of epithelial-derived cells—particularly in gland mucous cells, pit mucous cells, and malignant cells—as well as in a fraction of fibroblasts. Scattered cells with high AKT1 expression were also detected among CD8^+^ T cells and plasma cells (Figure 5G). Quantitative analysis of mean AKT1 expression across all cell types confirmed that gland mucous cells and malignant cells exhibited the highest average transcript levels, followed by pit mucous cells and mast cells, while immune cells such as CD8^+^ T cells, dendritic cells, and plasma cells showed the lowest mean expression (Figure 5H). These results indicate that AKT1 is primarily enriched in the epithelial/tumor compartment of GC, with limited expression in the immune microenvironment.

In summary, molecular docking identified AKT as a potential direct target of terfenadine. Multi-omics and single-cell analyses confirmed that AKT protein is broadly expressed across GC subtypes, is enriched in malignant cells, and its high expression predicts poor patient prognosis. Together, these findings support the hypothesis that terfenadine may exert its anti-proliferative effects in GC cells, at least in part, by targeting AKT.

### 3.6 Terfenadine modulated the expression of PI3K/AKT/mTOR pathway-associated proteins in GC AGS and HGC27 cells

**FIGURE 6:**
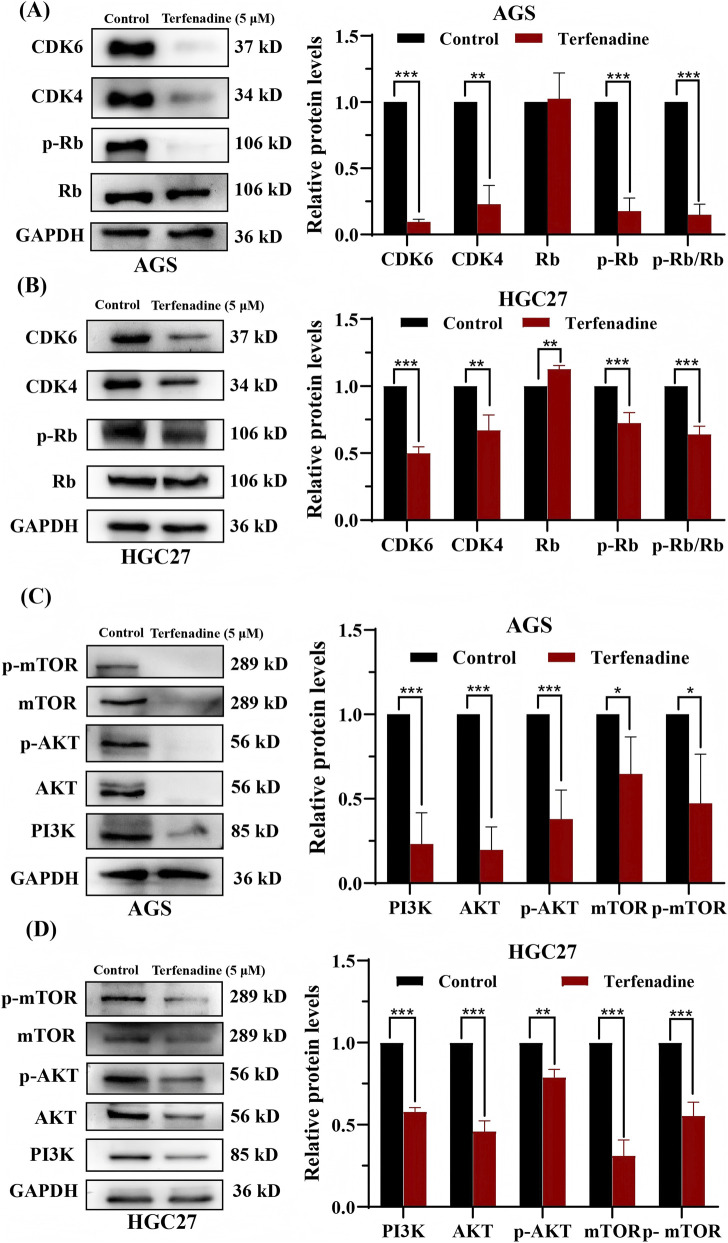
Terfenadine modulates the expression of cell cycle-related proteins and components of the PI3K/AKT/mTOR signaling pathway in GC cells. AGS and HGC27 cells were treated with terfenadine (5.00 μM) for 48 h, and protein expression was analyzed by Western blotting **(A,B)** Effects of terfenadine on cell cycle regulatory proteins in AGS **(A)** and HGC27 **(B)** cells. Terfenadine downregulated CDK4, CDK6, and phosphorylated Rb (p-Rb), while total Rb levels were relatively increased, resulting in a significantly decreased p-Rb/total Rb ratio **(C,D)** Effects of terfenadine on PI3K/AKT/mTOR pathway components in AGS **(C)** and HGC27 **(D)** cells. Terfenadine reduced the expression of PI3K, total AKT, phosphorylated AKT (p-AKT), total mTOR, and phosphorylated mTOR (p-mTOR). GAPDH served as a loading control. Representative blots from three independent experiments are shown.

We next examined the effect of terfenadine on the PI3K/AKT/mTOR signaling axis, a key regulator of cell survival and proliferation in GC. Western blot analysis revealed that terfenadine treatment (5 μM, 48 h) significantly downregulated CDK4, CDK6, and phosphorylated Rb (p-Rb) in both AGS and HGC27 cells (Figures 6A,B), consistent with the observed G0/G1 phase arrest (Figures 2A,B). Notably, total Rb protein levels were relatively increased, suggesting that terfenadine primarily affects Rb phosphorylation rather than its synthesis.

Importantly, terfenadine concurrently reduced the expression of multiple components of the PI3K/AKT/mTOR pathway. In AGS cells, the levels of PI3K, AKT, p-AKT, mTOR, and p-mTOR were all decreased compared to the control group (0.23 ± 0.18, 0.20 ± 0.14, 0.38 ± 0.17, 0.65 ± 0.22, 0.47 ± 0.29) (Figure 6C). Similar reductions were observed in HGC27 cells (PI3K: 0.58 ± 0.02, AKT: 0.46 ± 0.06, p-AKT: 0.79 ± 0.05, mTOR: 0.31 ± 0.10, p-mTOR: 0.55 ± 0.08) (Figure 6D).

It is noteworthy that both total and phosphorylated forms of these proteins were reduced to similar extents. Consequently, the calculated p-AKT/AKT and p-mTOR/mTOR ratios did not show statistically significant changes upon terfenadine treatment in either AGS or HGC27 cells, whereas the p-Rb/total Rb ratio was significantly decreased (Figures 6A,B). These observations suggest that the reduced phosphorylation of AKT and mTOR may be, at least in part, a consequence of decreased total protein abundance, rather than a direct inhibition of kinase activity. Whether this reflects a broader effect on protein synthesis or stability, or a specific feedback mechanism within the pathway, remains to be elucidated. Nevertheless, regardless of the precise mechanism, the consistent downregulation of these key signaling nodes, together with the functional outcomes (cell cycle arrest and apoptosis), indicates that the PI3K/AKT/mTOR axis is significantly affected by terfenadine treatment.

### 3.7 Terfenadine synergizes with 5-Fu to inhibit proliferation of gastric cancer cells

The combination of terfenadine and 5-Fu was then assessed using both Loewe and HSA models. In AGS cells, the Loewe model yielded an overall synergy score of 10.03 and an MSA score of 14.00, while the HSA model produced a synergy score of 11.73 and an MSA score of 17.05; both models thus classified the interaction as synergistic (scores >10). In HGC27 cells, the Loewe model generated an overall synergy score of 10.17 and an MSA score of 14.56, and the HSA model gave a synergy score of 11.04 with an MSA score of 20.16, again indicating synergy according to both reference models. Visual inspection of dose-response matrices, synergy heatmaps, and 3D surface plots confirmed that synergistic activity was concentrated in distinct concentration regions—terfenadine 0.50–1.25 μM combined with 5-Fu 1.25–2.50 μM in AGS cells, and terfenadine 1.25–2.50 μM combined with 5-Fu 2.50–5.00 μM in HGC27 cells—where observed inhibition reached 60%–80%, substantially exceeding the additive expectation from single-agent responses. At higher drug concentrations, inhibition plateaued and the interaction shifted toward additivity.

### 3.8 Terfenadine suppresses tumor growth in a GC PDX model

**FIGURE 7:**
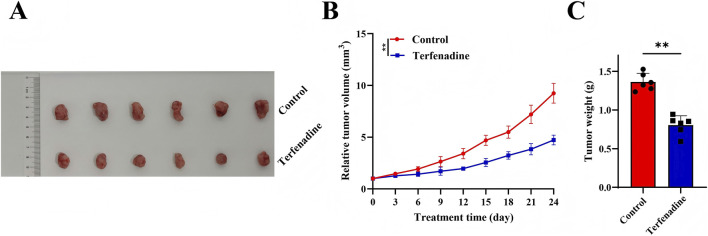
Terfenadine suppresses tumor growth in a gastric cancer patient-derived xenograft (PDX) model. Mice bearing P3 PDX tumors (initial volume ∼150 mm^3^) were randomly assigned to receive either vehicle (5% DMSO +30% PEG300 + 65% saline) or terfenadine (10 mg/kg, administered intraperitoneally once daily, n = 6 per group) for 24 days **(A)** Representative images of excised tumors from the vehicle control and terfenadine-treated groups at the end of the study. Scale bar, 1 cm **(B)** Tumor growth curves showing relative tumor volume over the 24-day treatment period. Data are presented as mean ± SD. Statistically significant differences between groups emerged from day 12 onward (*p* < 0.05 at day 12, *p* < 0.01 thereafter). At day 24, the mean relative tumor volume in the control group reached 9.23 ± 0.42, compared to 4.73 ± 0.20 in the terfenadine-treated group, representing a 48.7% reduction (*p* < 0.001) **(C)** Final tumor weights at the end of the study. The mean tumor weight was 1.36 ± 0.05 g in the control group *versus* 0.80 ± 0.05 g in the terfenadine group (*p* < 0.001). Statistical significance was determined by t-test.

The antitumor activity of terfenadine was assessed in the GC PDX model. As shown in Figure 7, terfenadine treatment significantly suppressed tumor growth compared to the control group throughout the 24-day observation period. At day 24, the mean relative tumor volume in the control group reached 9.23 ± 0.42, whereas in the terfenadine-treated group it was only 4.73 ± 0.20 (n = 6), representing a 48.7% reduction (*p* < 0.001). Statistically significant differences between the two groups emerged from day 12 onward (*p* < 0.05 at day 12, *p* < 0.01 thereafter). Consistent with the volume data, terfenadine treatment led to a marked decrease in final tumor weight: the mean tumor weight in the control group was 1.36 ± 0.05 g, compared to 0.80 ± 0.05 g in the terfenadine group (*p* < 0.001). These results demonstrate that terfenadine effectively inhibits tumor progression in a clinically relevant PDX model of GC.

## 4 Discussion

GC remains a significant contributor to cancer-related mortality worldwide (Bray et al., 2024), primarily because the majority of patients are diagnosed at advanced stages with poor prognosis and limited therapeutic options (Ma et al., 2023; Smyth et al., 2020; Rawla and Barsouk, 2019). Current chemotherapy regimens, while foundational, face limitations from systemic toxicity and multidrug resistance (MDR) (Jin et al., 2022; Liu et al., 2021). Drug repurposing of H1R antagonists like terfenadine offers a strategic approach to bypass *de novo* drug development barriers (Xia et al., 2024). Although H1R antagonists exhibited pleiotropic antitumor effects in CRC and BC (Baniya et al., 2024; Fernández-Nogueira et al., 2018), their role in GC remains underexplored.

Previous studies had predominantly focused on the therapeutic repurposing of H2 receptor (H2R) antagonists in GC management (Mohamad et al., 2024; Meng et al., 2023). However, epidemiological analyses revealed that prolonged H2R antagonist use correlated with increased risks of gastrointestinal malignancies, particularly GC (Meng et al., 2023). This historical emphasis on H2R antagonists contrasted with the under investigated yet promising role of H1R antagonists in modulating tumor-associated immune cells and angiogenesis, warranting systematic exploration in gastric malignancies (Bakhtiari et al., 2023; H et al., 2024; Trybus and Trybus, 2024; Zhao et al., 2020; Faustino-Rocha et al., 2017; González and Estes, 1998; Baniya et al., 2024; Fernández-Nogueira et al., 2018; Wang et al., 2014; Ellegaard et al., 2016; Nicolau-Galmés et al., 2011; Jangi et al., 2008; An et al., 2017). Our findings position terfenadine as an agent that modulates both PI3K/AKT/mTOR signaling and cell cycle regulators in GC, addressing critical therapeutic gaps.

The observed superior cytotoxicity of terfenadine compared to 5-Fu in GC cells may stem from multifaceted mechanisms and tumor-specific vulnerabilities. First, while 5-Fu primarily acts as an antimetabolite by inhibiting thymidylate synthase and disrupting DNA synthesis, terfenadine exerted pleiotropic anticancer effects beyond its classical anti-allergic role. Our studies suggested that terfenadine induced G0/G1 cell cycle arrest *via* downregulation of CDK4/6 and promoted apoptosis through mitochondrial pathway. Second, terfenadine may exhibit selective toxicity toward GC cells with specific molecular profiles, which could be attributed to intrinsic genetic mechanisms inherent to the cell lines, such as differential expression of H1R or epigenetic alterations. However, direct validation in normal gastric epithelial cells is required to confirm this selectivity. Third, terfenadine’s lipophilic nature may facilitate intracellular accumulation, contributing to its potent effects.

The PI3K/AKT/mTOR signaling pathway played a pivotal role in GC progression by regulating cell survival, proliferation, apoptosis, angiogenesis, and metastasis (Baghery Saghchy Khorasani et al., 2021; Morgos et al., 2024). Dysregulation of this pathway was closely associated with poor prognosis and therapeutic resistance in GC patients (Baghery Saghchy Khorasani et al., 2021). AKT, the central effector kinase of the PI3K pathway, is frequently hyperactivated in GC, with its enhanced activity clinically correlated with advanced GC grade and unfavorable prognosis (Kang and Chau, 2020; Song et al., 2019). Preclinical and clinical evidence strongly support the therapeutic potential of AKT inhibitions in GC management (Kang and Chau, 2020). Our study demonstrates that terfenadine suppresses GC cell proliferation by modulating the PI3K/AKT/mTOR signaling pathway, as evidenced by the reduced expression of key components including PI3K, AKT, and mTOR, as well as their phosphorylated forms.

While other GC cell lines (e.g., NCI-N87, SNU-1) are available, AGS, HGC27, and MKN45 were prioritized due to their extensive characterization in drug repurposing studies and consistent representation of PI3K/AKT/mTOR pathway alterations (Araújo et al., 2022; Fan et al., 2024; Yang et al., 2023). These lines collectively cover diverse GC subtypes (intestinal/diffuse) and molecular profiles, enhancing the generalizability of findings. The selection of 24-, 48-, and 72-h treatment windows aligns with established protocols in GC pharmacology and ensures comprehensive assessment of drug effects across multiple cellular processes (Araújo et al., 2022; Trybus and Trybus, 2024; Zhao et al., 2020; Baniya et al., 2024; Fernández-Nogueira et al., 2018; Wang et al., 2014; Ellegaard et al., 2016; Nicolau-Galmés et al., 2011; Jangi et al., 2008; An et al., 2017; Fan et al., 2024; Yang et al., 2023). This approach ensured methodological consistency with prior research while enabling robust evaluation of terfenadine’s therapeutic potential and mechanistic contributions (Araújo et al., 2022; Trybus and Trybus, 2024; Zhao et al., 2020; Baniya et al., 2024; Fernández-Nogueira et al., 2018; Wang et al., 2014; Ellegaard et al., 2016; Nicolau-Galmés et al., 2011; Jangi et al., 2008; An et al., 2017; Fan et al., 2024; Yang et al., 2023). Such standardization enhanced reproducibility and accelerates the translation of preclinical findings into clinical applications.

A key observation from our Western blot analyses is that terfenadine treatment reduced both total and phosphorylated levels of PI3K, AKT, and mTOR to similar extents, resulting in unchanged p-AKT/AKT and p-mTOR/mTOR ratios. This finding raises the possibility that the observed decrease in phosphorylation may be secondary to a decline in total protein abundance, rather than a specific inhibition of kinase activity. The reduction in total protein could stem from multiple mechanisms, including transcriptional repression, enhanced protein degradation, or a general inhibition of protein synthesis. While our data cannot definitively distinguish among these possibilities, the fact that GAPDH levels remained unchanged argues against a global, non-specific suppression of protein synthesis. However, to conclusively rule out a broader effect on translation, future studies should directly measure global protein synthesis rates (e.g., *via* puromycin incorporation or HPG assays) (Schmidt et al., 2009) and examine the phosphorylation status of direct mTORC1 downstream targets, such as S6K and 4E-BP1 (Burnett et al., 1998). Regardless of the precise mechanism, the consistent downregulation of PI3K/AKT/mTOR components and the associated functional consequences (cell cycle arrest and apoptosis) underscore the therapeutic relevance of targeting this axis in GC.

The interconnection between PI3K/AKT/mTOR suppression and mitochondrial apoptosis observed in our study is consistent with the established role of AKT as a master regulator of cell survival. AKT promotes cell survival through multiple mechanisms, including phosphorylation and inactivation of pro-apoptotic proteins (e.g., BAD, caspase-9), activation of anti-apoptotic proteins (e.g., Bcl-2, Bcl-xL), and regulation of transcription factors (e.g., FOXO, NF-κB) that control expression of survival genes (Baghery Saghchy Khorasani et al., 2021; Morgos et al., 2024; Kang and Chau, 2020; Song et al., 2019). Specifically, AKT-mediated phosphorylation of BAD at Ser136 prevents BAD from binding to and inhibiting anti-apoptotic Bcl-2 family members at the mitochondrial membrane, thereby preserving mitochondrial outer membrane potential and inhibiting cytochrome c release (Kang and Chau, 2020). Therefore, inhibition of AKT signaling by terfenadine likely relieves this brake, promoting BAD activation, mitochondrial permeabilization, and subsequent caspase activation. This mechanistic link is supported by our observations that terfenadine concurrently reduced p-AKT levels (Figures 6C,D) and induced loss of mitochondrial membrane potential (ΔΨm) (Figure 4B). Taken together, these findings indicate that PI3K/AKT/mTOR suppression and mitochondrial apoptosis are not independent events but rather mechanistically linked, with AKT inhibition serving as an upstream trigger for the mitochondrial apoptotic cascade.

**FIGURE 8:**
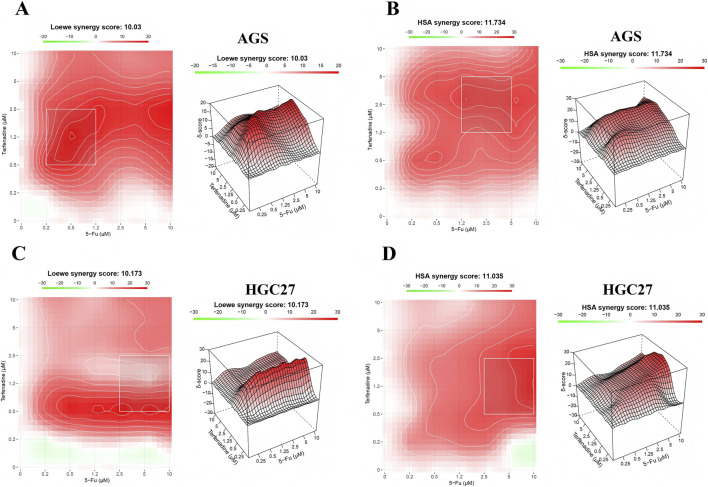
Terfenadine synergizes with 5-fluorouracil (5-Fu) to inhibit proliferation of gastric cancer cells. AGS and HGC27 cells were treated with serial dilutions of terfenadine (0–10.00 μM), 5-Fu (0–10.00 μM), or their combinations for 48 h. Cell viability was measured by MTT assay, and drug synergy was analyzed using SynergyFinder 3.0 with both Loewe and Highest Single Agent (HSA) reference models. Synergy scores were interpreted according to platform guidelines (scores < −10: antagonism, −10 to 10: additivity, >10: synergy). **(A, B)** Synergy analysis of terfenadine combined with 5-Fu in AGS cells **(A)** Loewe model: synergy heatmap (left) and 3D synergy surface plot (right) **(B)** HSA model: synergy heatmap (left) and 3D synergy surface plot (right) **(C,D)** Synergy analysis of terfenadine combined with 5-Fu in HGC27 cells **(C)** Loewe model: synergy heatmap (left) and 3D synergy surface plot (right) **(D)** HSA model: synergy heatmap (left) and 3D synergy surface plot (right). In AGS cells, the overall synergy scores were 10.03 (Loewe) and 11.73 (HSA), with most synergistic area (MSA) scores of 14.00 and 17.05, respectively. In HGC27 cells, the overall synergy scores were 10.17 (Loewe) and 11.04 (HSA), with MSA scores of 14.56 and 20.16, respectively. Synergistic activity was concentrated in distinct concentration regions: terfenadine 0.50–1.25 μM combined with 5-Fu 1.25–2.50 μM in AGS cells, and terfenadine 1.25–2.50 μM combined with 5-Fu 2.50–5.00 μM in HGC27 cells, where observed inhibition reached 60%–80%. Data represent three independent experiments with three technical replicates each.

The convergent mechanisms of 5-Fu-induced DNA damage and terfenadine-triggered mitochondrial apoptosis provide a plausible biological basis for the observed synergy in our combination studies (Figure 8). As an antimetabolite, 5-Fu exerts its primary cytotoxic effect through incorporation into DNA and RNA, leading to DNA damage and replication stress (Longley et al., 2003). This activates p53-dependent and -independent DNA damage responses (Ciccia and Elledge, 2010). However, cancer cells often evade 5-Fu-induced apoptosis through upregulation of survival pathways, including the PI3K/AKT/mTOR cascade (Baghery Saghchy Khorasani et al., 2021; Morgos et al., 2024). By suppressing this pathway, terfenadine may lower the apoptotic threshold, rendering cells more susceptible to 5-Fu-induced DNA damage. Additionally, the distinct cell cycle effects of the two agents—terfenadine-induced G0/G1 arrest (Figures 2A,B) and 5-Fu-induced S phase accumulation (Figures 2A,B)—may create schedule-dependent synergy. Terfenadine could first arrest cells in G0/G1, and upon release, cells may enter S phase synchronously, becoming more vulnerable to 5-Fu. Alternatively, the combination may trap cells in incompatible cell cycle states, promoting mitotic catastrophe. Notably, terfenadine exhibited superior pro-apoptotic activity in HGC27 cells, which are relatively resistant to 5-Fu (IC_50_ = 77.78 μM). This suggests that terfenadine could be particularly valuable in treating 5-Fu-resistant tumors, where its ability to suppress AKT signaling and induce mitochondrial apoptosis may bypass resistance mechanisms.

Beyond 5-Fu, the dual mechanism of terfenadine—targeting both PI3K/AKT/mTOR signaling and mitochondrial integrity—suggests potential synergy with other classes of chemotherapeutics, including platinum agents (e.g., cisplatin, oxaliplatin) and taxanes (e.g., paclitaxel, docetaxel). Future studies should systematically evaluate these combinations to optimize therapeutic strategies for GC.

In our study, terfenadine exerted concentration- and time-dependent cytotoxic effects in GC AGS and HGC27 cells, consistent with its previously reported activity in CRC HCT116 cells (Baniya et al., 2024). Flow cytometry demonstrated that terfenadine induced G0/G1 phase arrest in AGS (*p* < 0.05) and HGC27 cells (*p* < 0.01). This growth suppression aligned with terfenadine-induced G0/G1 phase arrest and apoptosis observed across multiple cancer types (Liu et al., 2003). Terfenadine treatment recapitulated the anti-migratory and anti-invasive effects of H1HR knockdown. Specifically, terfenadine impaired the migration and invasion of AGS and HGC27 GC cells (Figures 3C,D), paralleling the findings in H1HR-knockdown SNU-368 HCC cells (Zhao et al., 2020). This consistency across different cellular contexts and intervention methods underscores the critical role of H1R/H1HR in driving malignant phenotypes. Consistent with our Western blot results, terfenadine treatment in GC cells reduced the expression of key components of the PI3K/AKT/mTOR pathway, including both total and phosphorylated AKT, replicating a mechanism previously reported in MDA-MB-231 basal BC cells (Fernández-Nogueira et al., 2018).

It is worth noting that the percentage of apoptotic cells detected by Annexin V/PI staining at 48 h (∼70% in HGC27 cells at 5 μM) was higher than the corresponding reduction in cell viability measured by MTT assay (∼50%). This apparent discrepancy can be attributed to the different principles of the two assays. The MTT assay measures mitochondrial dehydrogenase activity as a surrogate for metabolically active cells, whereas Annexin V/PI staining directly detects phosphatidylserine externalization and membrane permeability as markers of apoptosis (Mosmann, 1983). Cells that have already committed to apoptosis (Annexin V^+^) may still retain residual mitochondrial activity, contributing to the MTT signal (Vermes et al., 1995). Moreover, at 48 h, many cells have entered late apoptosis or secondary necrosis and are unequivocally counted as apoptotic by flow cytometry, yet they may generate a weak MTT signal due to incomplete mitochondrial collapse. The two assays thus measure related but distinct biological processes, and the observed numerical difference reflects the complexity of cell death dynamics rather than a contradiction. These complementary assays together provide a more comprehensive picture of terfenadine-induced cell death.

While our molecular docking and Western blot data suggest that AKT is a plausible direct target of terfenadine, we acknowledge that definitive proof of direct targeting requires additional validation. Specifically, *in vitro* kinase activity assays using purified AKT protein would directly determine whether terfenadine inhibits AKT enzymatic function (Fabian et al., 2005). Mutational analysis of predicted binding residues (e.g., Asn54, Gln79) followed by functional studies in cells overexpressing wild-type *versus* mutant AKT would further substantiate the binding model and establish causality (Zhao et al., 2020). Additionally, techniques such as surface plasmon resonance or cellular thermal shift assays could provide orthogonal evidence of direct physical interaction (Martinez Molina et al., 2013). These experiments represent important future directions that will be pursued to fully elucidate the molecular mechanism of terfenadine.

The significant antitumor efficacy of terfenadine in the GC PDX model aligns with our bioinformatics findings that high AKT expression predicts poor prognosis (Figure 5E) and that AKT is enriched in malignant cells (Figures 5F–H). While we did not directly measure AKT expression in the PDX tumors used for efficacy studies, the observed growth inhibition (41.2% reduction in final tumor weight, *p* < 0.001) is consistent with the hypothesis that AKT-driven tumors may be particularly sensitive to terfenadine. Future studies should stratify PDX models based on baseline AKT expression or phosphorylation status to determine whether these biomarkers predict response to terfenadine. Such pharmacodynamic analyses would directly test whether the prognostic value of AKT expression (i.e., high AKT predicting poor prognosis) translates into predictive value (i.e., high AKT tumors responding better to terfenadine).

Based on our findings, we propose a preliminary stratification strategy for future clinical evaluation of terfenadine. AKT protein expression assessed by IHC could identify patients with AKT-high tumors who may benefit from terfenadine therapy. Given that AKT activity is regulated by phosphorylation, assessing p-AKT levels may provide a more dynamic measure of pathway activation and serve as a pharmacodynamic biomarker in early-phase trials (Yap et al., 2011). Furthermore, our observation that terfenadine induces G0/G1 arrest independent of p53 status—contrasting with the p53-dependent S phase arrest induced by 5-Fu—suggests that terfenadine may be particularly valuable in p53-mutant tumors, which are often resistant to conventional chemotherapy (Vogelstein et al., 2000). Prospective biomarker-driven trials are needed to validate these hypotheses and establish the optimal strategy for integrating terfenadine into GC treatment regimens.

The well-documented cardiotoxicity of terfenadine, primarily attributable to hERG channel blockade leading to QT prolongation, remains a significant barrier to its clinical repurposing. However, several strategies could potentially uncouple efficacy from cardiac risk. First, dose optimization through combination therapy offers a practical approach: our synergy data demonstrate that terfenadine synergizes with 5-Fu at concentrations (2.50–5.00 μM) below those associated with significant hERG blockade in preclinical models (typically >10 μM) (Olasińska-Wiśniewska et al., 2014). This suggests that by using terfenadine in combination with 5-Fu, it may be possible to achieve therapeutic antitumor effects at doses that are cardiotoxically safe. Furthermore, the concentration windows identified in our synergy analysis could guide the design of intermittent dosing schedules that maintain efficacy while allowing for cardiac recovery between doses. Clinical pharmacokinetic studies of terfenadine from its original use as an antihistamine established that plasma concentrations in the 0.50–2.50 μM range are achievable and well-tolerated; our synergy data suggest that even these lower concentrations, when combined with 5-Fu, may retain antitumor activity.

Second, nanocarrier-mediated tumor targeting offers a powerful approach to spatially separate efficacy from toxicity. By encapsulating terfenadine in tumor-targeted nanocarriers, it may be possible to achieve high intratumoral drug concentrations while minimizing peak plasma levels and cardiac exposure. Several studies have already explored this concept for terfenadine: for example, Baniya et al. (2024) demonstrated that terfenadine-loaded nanoparticles effectively suppressed colorectal cancer xenograft growth while reducing systemic toxicity (Baniya et al., 2024). Similarly, Fan et al. (2023) reviewed the potential of various nanocarrier systems—including liposomes, polymeric nanoparticles, and micelles—for delivering cardiotoxic drugs safely (Fan et al., 2023). In the context of GC, nanoparticle formulations could exploit the enhanced permeability and retention (EPR) effect or active targeting strategies to selectively deliver terfenadine to tumor tissue. Future preclinical development of terfenadine for GC should prioritize evaluation of such nanocarrier formulations.

Third, structural modification offers a longer-term strategy. Structure-activity relationship studies have identified key molecular features responsible for hERG binding—notably the tertiary amine and lipophilic aromatic groups. Medicinal chemistry efforts could modify these moieties while preserving interactions with AKT (e.g., hydrogen bonding with Asn54 and Gln79, π-π stacking with Trp80, as identified in our docking studies). For instance, fexofenadine, the active metabolite of terfenadine, was developed specifically to eliminate cardiotoxicity and is now a widely used, safe antihistamine. Interestingly, fexofenadine has also shown some anticancer activity in preclinical studies, raising the possibility that further optimization could yield a compound with both enhanced safety and efficacy. Our molecular docking data could guide rational design of safer terfenadine derivatives that maintain antitumor activity but lack hERG affinity.

Another important consideration for clinical translation is the potential selectivity of terfenadine toward cancer cells *versus* normal cells. In this study, we did not include a normal gastric epithelial cell line (e.g., GES-1) as a control in our cytotoxicity assays, which is a limitation of our work. While our findings demonstrate potent antitumor effects in GC cells, the selectivity of these effects remains to be determined. Although direct comparative data in gastric cells are lacking, previous studies in other cancer types suggest that terfenadine may exhibit selective cytotoxicity toward malignant cells. As reviewed by Trybus and Trybus (2024), diphenhydramine, triprolidine, astemizole, and terfenadine have been shown to induce apoptosis in multiple human melanoma cell lines while demonstrating minimal toxicity toward normal melanocytes and mouse embryonic fibroblasts. This body of evidence indicates that certain antihistamines, including terfenadine, may preferentially affect cancer cells while sparing normal counterparts. Future studies should include comparative cytotoxicity assays in GES-1 or other non-cancerous gastric cell lines to establish the therapeutic window of terfenadine.

Despite demonstrating the efficacy of terfenadine in suppressing AKT signaling and impairing malignant phenotypes in GC cells, our study has several limitations that outline clear paths for future investigation. Foremost, the precise molecular targets of terfenadine remain incompletely defined. While our molecular docking studies identified AKT as a plausible direct target (docking score = −9.09) and Western blot analysis confirmed downregulation of PI3K/AKT/mTOR pathway components, the concurrent reduction of both total and phosphorylated forms of these proteins (resulting in unchanged p-AKT/AKT and p-mTOR/mTOR ratios) suggests that the observed effects may not stem from direct kinase inhibition. This mechanistic ambiguity is clinically relevant, given the limited efficacy and toxicity (e.g., hyperglycemia, neutropenia, diarrhea) observed with direct AKT inhibitors in GC trials (Kang and Chau, 2020). Therefore, we will use proteomic profiling and genome-wide CRISPR-Cas9 screens to identify terfenadine’s direct binding partners and genetic modifiers, alongside using H1R-specific tools to definitively determine its dependency on H1R.

Second, although we have demonstrated potent synergistic cytotoxicity between terfenadine and 5-Fu in 2 GC cell lines (AGS and HGC27) using rigorous checkerboard assays and two reference models (Loewe and HSA), the mechanistic basis of this synergy remains to be fully elucidated. The distinct concentration windows identified—0.50–1.25 μM terfenadine combined with 1.25–2.50 μM 5-Fu in AGS cells, and 1.25–2.50 μM terfenadine combined with 2.50–5.00 μM 5-Fu in HGC27 cells—provide a foundation for future mechanistic studies. Whether the synergy arises from terfenadine-mediated lowering of the apoptotic threshold, schedule-dependent cell cycle interactions (G0/G1 arrest vs. S phase accumulation), or other mechanisms requires further investigation through time-course and sequential treatment studies.

Third, while we have provided the *in vivo* evidence of terfenadine’s antitumor efficacy in a GC PDX model (48.7% reduction in relative tumor volume and 41.2% reduction in final tumor weight, *p* < 0.001), this represents a single model with limited sample size. The antitumor activity across the full spectrum of molecular GC subtypes—including MSI-high, EBV-positive, and genomically stable tumors—remains unknown. Future work must evaluate terfenadine in a broader panel of PDX models representing key molecular subtypes and incorporate pharmacodynamic analyses to correlate tumor response with baseline AKT expression or phosphorylation status. Such studies would directly test whether the prognostic value of high AKT expression (HR = 2.33, *p* < 1E-16) translates into predictive value for terfenadine response.

Finally, as with many repurposed agents, the therapeutic window of terfenadine requires rigorous definition. While our synergy data suggest that clinically achievable, well-tolerated concentrations (0.50–2.50 μM) may retain antitumor activity when combined with 5-Fu, and while our PDX study demonstrated *in vivo* efficacy without overt toxicity, we did not include a normal gastric epithelial cell line (e.g., GES-1) as a control in our cytotoxicity assays. Future studies must systematically evaluate terfenadine’s selectivity through comparative cytotoxicity assays in GES-1 or other non-cancerous gastric cell lines, and incorporate comprehensive toxicological assessments in *vivo* models to establish its therapeutic index. In summary, addressing these limitations—from defining the molecular mechanism and mechanistic basis of synergy to validating efficacy across diverse molecular subtypes and establishing therapeutic selectivity—will be crucial to fully delineate terfenadine’s translational potential in GC.

## 5 Conclusion

In this study, we demonstrate that terfenadine exerts robust antitumor activity against GC through multifaceted mechanisms, with the PI3K/AKT/mTOR signaling pathway identified as a key node affected by the drug.

Molecular docking analyses predicted that terfenadine binds to AKT with high affinity (docking score = −9.09), forming potential hydrogen bonds with Asn54 and Gln79, as well as hydrophobic and electrostatic interactions with multiple surrounding residues (Figures 5A,B). These *in silico* findings suggest that AKT is a plausible direct target of terfenadine, although experimental validation is required.

The clinical relevance of AKT in GC was substantiated by multi-omics and single-cell analyses. AKT protein expression was independent of MSI status (Figure 5C), and although AKT1 mRNA levels showed no significant difference between tumor and normal tissues (Figure 5D)---suggesting predominant post-transcriptional regulation---high AKT1 expression was strongly associated with poor patient prognosis (HR = 2.33, *p* < 1E-16, Figure 5E), underscoring its clinical significance. Single-cell transcriptomic profiling further refined the cellular context of AKT expression, confirming its enrichment in malignant epithelial cells (Figures 5F–H), particularly in gland mucous cells and malignant clusters, with only limited expression in immune and stromal compartments. This expression pattern confirms that the AKT pathway is primarily active within the tumor epithelium, providing a direct rationale for targeting it in cancer cells.

Functionally, terfenadine treatment in GC cells resulted in G0/G1 phase cell cycle arrest (mediated by downregulation of CDK4, CDK6, and phosphorylated Rb), induction of mitochondrial apoptosis, and suppression of migration. At the molecular level, terfenadine treatment was associated with reduced expression of multiple components of the PI3K/AKT/mTOR cascade, including PI3K, AKT, p-AKT, mTOR, and p-mTOR. Notably, both total and phosphorylated forms of these proteins were decreased to similar extents, suggesting that the observed reduction in phosphorylation may reflect, at least in part, a decrease in total protein abundance. Whether this results from effects on protein synthesis, stability, or transcriptional regulation, or represents a broader cellular response, remains to be elucidated.

Importantly, this study further demonstrates that the combination of terfenadine and 5-Fu exerts strong synergistic cytotoxicity in both AGS and HGC27 GC cells, as consistently quantified by Loewe and HSA models with overall synergy scores exceeding 10 and MSA scores reaching up to 20.16. This synergistic interaction is confined to distinct, clinically achievable concentration windows, enabling substantial dose reduction of both agents—particularly 5-Fu—which may help mitigate dose-limiting toxicities and overcome chemoresistance. The convergent mechanisms of 5-Fu-induced DNA damage and terfenadine-triggered mitochondrial apoptosis *via* hERG channel blockade and calcium overload provide a plausible biological basis for the observed synergy.

The *in vivo* antitumor efficacy of terfenadine was further validated in a GC PDX model. Terfenadine treatment significantly reduced tumor growth rate and decreased final tumor weight compared to control groups, providing direct preclinical evidence of its therapeutic potential in a clinically relevant model.

Notably, terfenadine exhibited superior pro-apoptotic activity compared to 5-Fu, particularly in 5-Fu-resistant HGC27 cells, and induced G0/G1 arrest independent of p53 status—contrasting with the p53-dependent S phase arrest induced by 5-Fu. These findings further support the repurposing of terfenadine as an effective alternative or adjunct to conventional chemotherapy in GC.

In summary, this study provides evidence that terfenadine exerts anti-GC effects associated with modulation of the PI3K/AKT/mTOR pathway. The clinical relevance of AKT is supported by its frequent expression in malignant cells and its strong association with poor prognosis. Furthermore, the potent synergistic interaction between terfenadine and 5-Fu, combined with its *in vivo* efficacy in a PDX model, positions terfenadine as an accessible, low-cost adjunct to 5-Fu-based chemotherapy for GC. These findings support the repurposing of terfenadine and underscore the importance of employing multiple reference models to robustly quantify drug synergy. While AKT represents a promising therapeutic target based on our preclinical findings, its predictive value for patient stratification and response to terfenadine requires validation in prospective biomarker-driven trials. Given its established safety profile and the observed biological activities presented here, terfenadine represents a promising multi-targeted agent worthy of further investigation to elucidate its precise molecular mechanisms and evaluate its clinical applicability in GC treatment regimens.

## Data Availability

The original contributions presented in the study are included in the article/Supplementary Material, further inquiries can be directed to the corresponding authors.
