# Supplementary material for: Antihistamine drug terfenadine suppressed the cycle progression of gastric cancer cells by targeting PI3K/AKT/mTOR signaling
Source: Front Pharmacol. 2026 Mar 31;17:1723444. doi: 10.3389/fphar.2026.1723444 (PMC13076520; doi:10.3389/fphar.2026.1723444)
Supplement: Supplementary file 1 [file Table1.docx]

Supplementary Material

# Supplementary Table

Supplementary Table S1. Molecular docking scores of terfenadine with candidate proteins. The table lists the protein targets (with PDB IDs), their primary functions, and the corresponding molecular docking scores obtained from Schrödinger software. The result highlights the strong binding affinity between terfenadine and AKT.

**Table S1**. The molecular docking scoring of terfenadine and proteins

| Target | PDB ID | Resolution(Å) | Organisms | Docking score |
| --- | --- | --- | --- | --- |
| AKT | 6hhf | 2.9 | *Home sapiens* | -9.08743 |
| MEK | 5eym | 2.7 | *Home sapiens* | -7.89504 |
| Muscarinic acetylcholine receptor M1 | 5cxv | 2.7 | *Home sapiens* | -7.75444 |
| CDK6 | 4ez5 | 2.7 | *Home sapiens* | -7.49977 |
| Bcl-2 | 6qgg | 1.5 | *Home sapiens* | -7.10601 |
| CDK7 | 8p4z | 2.75 | *Home sapiens* | -6.86319 |
| JAK | 5tq6 | 2.06 | *Home sapiens* | -6.78056 |
| Bcl-xl | 7lh7 | 1.409 | *Home sapiens* | -6.48887 |
| JNK | 4y5h | 2.055 | *Home sapiens* | -6.48349 |
| CyclinE1 | 5l2w | 2.8 | *Home sapiens* | -6.39033 |
| Raf | 5fd2 | 2.89 | *Home sapiens* | -6.07459 |
| mTOR | 4jt6 | 3.6 | *Home sapiens* | -6.07429 |
| PARP | 6bhv | 2.3 | *Home sapiens* | -5.96534 |
| Erk5 | 6hkn | 2.33 | *Home sapiens* | -5.85422 |
| caspase-3 | 3dej | 2.6 | *Home sapiens* | -5.81444 |
| CyclinA | 6gue | 1.99 | *Home sapiens* | -5.71761 |
| CDK2 | 5nev | 2.97 | *Home sapiens* | -5.71409 |
| CDK1 | 6gu2 | 2 | *Home sapiens* | -5.58393 |
| CyclinD1 | 5vzu | 2.7 | *Home sapiens* | -5.53216 |
| MAPK | 2g01 | 3.5 | *Home sapiens* | -5.25833 |
| PI3K | 6gvf | 2.5 | *Home sapiens* | -4.9919 |
| E-cadherin | 4zte | 2.13 | *Home sapiens* | -4.42986 |
| STAT3 | 6nuq | 3.15 | *Home sapiens* | -4.17938 |
| caspase-9 | 1nw9 | 2.4 | *Home sapiens* | -4.10059 |
| GSK-3β | 1pyx | 2.4 | *Home sapiens* | -4.04215 |
| NF-kB | 1a3q | 2.1 | *Home sapiens* | -3.67760 |
| β-catenin | 7afw | 1.81 | *Home sapiens* | -3.33701 |
| Wnt | 6gbi | 1.25 | *Home sapiens* | -3.27329 |
| Ras | 7jhp | 2.766 | *Home sapiens* | -3.00611 |
| Histamine H1 Receptor | 3rze | 3.1 | *Home sapiens* | -2.08368 |
